# Effect of Statins and Renin–Angiotensin–Aldosterone System Inhibitors on IL-6 Levels in COVID-19 Patients

**DOI:** 10.3390/jcm13216414

**Published:** 2024-10-26

**Authors:** Laura Pereckaite, Neringa Vaguliene, Agne Vitkauskaite, Astra Vitkauskiene, Daiva Urboniene

**Affiliations:** 1 Department of Laboratory Medicine, Faculty of Medicine, Medical Academy, Lithuanian University of Health Sciences, Eiveniu Str. 2, LT-50161 Kaunas, Lithuania; 2 Department of Pulmonology, Faculty of Medicine, Medical Academy, Lithuanian University of Health Sciences, Eiveniu Str. 2, LT-50161 Kaunas, Lithuania; 3 Department of Obstetrics and Gynecology, Faculty of Medicine, Medical Academy, Lithuanian University of Health Sciences, Eiveniu Str. 2, LT-50161 Kaunas, Lithuania

**Keywords:** COVID-19, SARS-CoV-2, cardiovascular co-morbidities, medication, statins, renin–angiotensin–aldosterone system inhibitors, IL-6

## Abstract

**Background/Objectives:** Severe clinical course and mortality from COVID-19 are mostly associated with increased concentrations of IL-6 and IL-10. Findings from clinical trials suggest that both statins and renin–angiotensin–aldosterone system inhibitors (RAASI) might have the potential to reduce unfavorable outcomes in patients with COVID-19. The aim of this study was to evaluate the effect of statins and RAASI on the cytokine concentrations in COVID-19 patients. **Methods:** SARS-CoV-2 infected patients were enrolled in this study, and demographic, clinical, and routine laboratory data were evaluated. Plasma cytokine levels were measured by multiplex assay. **Results:** COVID-19 patients with chronic cardiovascular diseases (CVD) had significantly lower median plasma IL-6 levels than COVID-19 patients with no co-morbidities (26 vs. 53 pg/mL, *p* = 0.021). COVID-19 patients with CVD who were taking statins had significantly lower median concentrations of IL-6 (21 vs. 44 pg/mL, *p* = 0.027), TNFα (21 vs. 39.5 pg/mL, *p* = 0.036), and IL-10 (19 vs. 25.5 pg/mL, *p* = 0.025) compared to COVID-19 patients with no CVD. In a binary logistic regression model, IL-6 was a significant variable, with an odds ratio value of 0.961 (95% CI 0.929–0.995). Regarding RAASI, only plasma IL-6 (22 vs. 44 pg/mL, *p* = 0.012) levels were found to be significantly lower in COVID-19 patients with CVD consuming these medications compared to patients who did not have any CVD. **Conclusions:** COVID-19 patients who had chronic cardiovascular co-morbidities and who were administered statins or RAASI had significantly lower concentrations of IL-6 than COVID-19 patients who did not have any co-morbidities. These findings suggest that the use of statins or RAASI may be of value in COVID-19 patients.

## 1. Introduction

Coronavirus disease 2019 (COVID-19), caused by the severe acute respiratory syndrome coronavirus 2 (SARS-CoV-2), is known for its distinctive and unpredictable clinical course [1]. The severity of COVID-19 mostly depends on the interaction between SARS-CoV-2 virulence factors and the genetic as well as immune factors of the host [2]. In retrospect, it has become clear that infection was particularly dangerous for patients of advanced age, with cardiovascular diseases, obesity, diabetes, chronic respiratory disease, cancer, or suppressed immune functions [3].

Severe clinical courses of COVID-19 and patient mortalities were mainly caused by uncontrolled immune reactions and abnormal inflammatory responses, resulting in the secretion of large amounts of cytokines. Cytokines produced by different cells during physiological and pathological processes transfer the signals between cells, and in cases of infections, regulate local and systemic inflammatory responses [2]. Normally, the secretion of cytokines and chemokines confers an effective innate and adaptive immune response to clear the infectious agent and prevent re-infection [4]. According to the literature, unfavorable outcomes of COVID-19 disease were found to be mostly associated with increased concentrations of interleukin (IL)-6 and IL-10 [5].

Several meta-analyses of clinical trials suggest that both statins and renin–angiotensin–aldosterone system inhibitors (RAASI) have the potential to reduce unfavorable outcomes in patients with COVID-19 [6,7]. Statins are a well-known group of cholesterol-lowering medicines, and their anti-inflammatory effects have been reported in some studies. Using statins, inflammation was successfully reduced in patients with metabolic syndrome, polycystic ovarian syndrome, and renal dialysis [8]. The anti-inflammatory, immunomodulatory, and antithrombotic properties of statins might also be useful in COVID-19 as it is associated with high-grade inflammation [9]. According to study data, statins inhibit the synthesis of IL-6 and C-reactive protein; they also effectively protect against vascular injury and subsequent internal organ damage [8,9]. Despite the conflicting literature data, another cardiovascular medication group, RAASI, are noted for anti-inflammatory and immunomodulatory action [9]. Renin–angiotensin–aldosterone system dysregulation is an important mechanism in several co-morbidities that increase the susceptibility to severe COVID-19, including hypertension and diabetes. Therefore, RAASI are considered first-line agents to decrease cytokine production and to diminish end-organ damage in mentioned conditions [10].

Based on these findings in the literature, the aim of this study was to evaluate the effect of statins and RAASI on cytokine concentrations in COVID-19 patients. This study also sought to determine to what extent a co-morbidity such as chronic cardiovascular disease (CVD) could contribute to any untoward outcomes due to the presence of either of these drugs in a COVID-19 patient’s system.

## 2. Materials and Methods

### 2.1. Study Participants

This study was conducted according to the guidelines of the Declaration of Helsinki and approved by the Kaunas Regional Biomedical Research Ethics Committee. Participation criteria included adult patients with polymerase chain reaction (PCR)-confirmed SARS-CoV-2 infection who were admitted to the Hospital of Lithuanian University of Health Sciences Kauno klinikos (HLUHS KK) for treatment of COVID-19 disease. Patients in this study were enrolled from December 2020 to February 2022. Fifteen healthy volunteers were also included as a control group. Each enrolled individual provided written consent to participate in this study.

According to the criteria published by local and international authorities including the National Institutes of Health (NIH) and World Health Organization (WHO), patients with COVID-19 were assigned to one of the four clinical severity groups as shown in Table 1 [11,12].

### 2.2. Study Data

Demographic, clinical, and routine laboratory test data of the participating patients were collected. For the analysis of cytokines, venous blood samples were collected into vacuum EDTA-coated tubes; plasma was separated immediately and stored at −80 °C until analysis. The concentrations (pg/mL) of interferon (IFN)β, IFNγ, IL-1α/IL-F1, IL-6, IL-12/IL-23p40, IL-2, IL-10, tumor necrosis factor (TNF)α, and lipocalin-2/neutrophil gelatinase-associated lipocalin (NGAL) were measured using a commercially available magnetic bead-based multiplex Human Pre-mixed Multi-Analyte Kit Luminex Assay kit (R&D Systems, Minneapolis, MN, USA) in association with a Luminex 200 analyzer (Invitrogen, San Jose, CA, USA). The level of sensitivity of the kit was IFNβ 13.95 pg/mL, IFNγ 54.32 pg/mL, IL-1α/IL-F1 4.81 pg/mL, IL-6 4.4 pg/mL, IL-12/IL-23p40 526.54 pg/mL, IL-2 28.77 pg/mL, IL-10 4.03 pg/mL, TNFα 7.53 pg/mL, and lipocalin-2/NGAL 122.92 pg /mL.

All sample preparations and analyses were performed at the Department of Laboratory Medicine, HLUHS KK. Health and safety precautions were complied with in all laboratory work (according to HLUHS KK regulations) and user manuals for the reagents and equipment.

### 2.3. Statistical Analysis

All collected data were stored in electronic spreadsheets. Statistical analysis of the data was performed using SPSS Statistics 29 software (IBM, Chicago, IL, USA). Distribution of the quantitative data was checked using Kolmogorov–Smirnov and Shapiro–Wilk tests. Normally distributed quantitative data were reported as means and standard deviation (SD). Non-normally distributed quantitative data were reported using medians, as well as first (Q_1_) and third (Q_3_) quartiles. The distributions of these data in the independent samples were compared using non-parametric Mann–Whitney U and Kruskal–Wallis tests. Post hoc pair-wise comparisons with Bonferroni’s correction were performed where necessary. Qualitative data were reported using frequencies (*n, N*) and percentages (%). To detect the associations between the data, the Chi-square (Χ^2^) test of independence or Fisher’s exact test for small samples (*n* ≤ 5) were used. Pair-wise comparisons between the groups were performed using a z-test with Bonferroni’s correction. A binary logistic regression model was applied to investigate the effects between the variables using the odds ratio (OR) and 95% confidence interval (CI). All statistically significant findings were assumed to have a *p*-value < 0.05 and a 95% CI not containing the values 0 or 1.

## 3. Results

### 3.1. Characteristics of COVID-19 Patients

A total of 106 COVID-19 patients were enrolled in this study. The median time from symptom onset to hospitalization was 6 (Q_1_ = 1, Q_3_ = 10) days. Seventy (66.04%) of the studied patients were males. The mean age of the patients was 58.59 (SD = 14.92) years. Thirty-eight (35.85%) of the patients were 65 years and older. Only 13 (12.26%) of the patients were vaccinated against COVID-19 disease. Ten of them were completely vaccinated with two or three vaccine doses. Seven fully vaccinated patients received Comirnaty (BioNTech Manufacturing, Mainz, Germany), and three received Vaxzevria (AstraZeneca, Cambridge, Sweden) vaccines. The mean time between the onset of SARS-CoV-2 infection symptoms and blood sampling was 9 days (Q_1_ = 5, Q_3_ = 12 days).

According to clinical and radiologic imaging studies, all COVID-19 patients were divided into four distinct severity groups as shown in Table 2.

In this patient cohort, critical COVID-19 was associated with a significant increase in IL-6 concentration (*p* = 0.009). The median IL-6 concentration in critical COVID-19 was 55 pg/mL (Q_1_ = 31 pg/mL, Q_3_ = 120.88 pg/mL), while in non-critical COVID-19 the median concentration was 27.75 pg/mL (Q_1_ = 17 pg/mL, Q_3_ = 55.38 pg/mL). Seventeen study patients (16.04%) did not survive the COVID-19 disease. Most of them, 14 (82.35%, Fisher’s exact test *p* < 0.001), belonged to the critical COVID-19 group. The deceased patients had significantly higher concentrations of IL-6 and IL-10 compared to recovered patients (Table 3).

However, the deceased COVID-19 patients were not statistically significantly different from COVID-19 patients who survived in regards to their age, sex, vaccination status, co-morbidities (including chronic cardiovascular diseases), and regularly consumed medications (including statins and RAASI).

Patients given remdesivir for specific treatment of SARS-CoV-2 infection did not have statistically different concentrations of IL-6 (*p* = 0.563) or IL-10 (*p* = 0.292). However, patients with dexamethasone and tocilizumab administration had significantly higher concentrations of IL-6 and IL-10 compared to those who did not require these medications (Table 4).

Dexamethasone and tocilizumab were mostly administered in COVID-19 patients with severe and critical disease. A total of 94.92% (*n* = 56) of severe and 100% (*n* = 14) of critical COVID-19 patients were given dexamethasone versus 44.44% (*n* = 12) of patients with moderate COVID-19 disease (Pearson’s Χ^2^ = 51.681; *p* < 0.001). Patients with mild COVID-19 did not receive dexamethasone. In total, 11.86% (*n* = 7) of severe and 35.71% (*n* = 5) of critical COVID-19 patients were given tocilizumab, while none of the patients with mild and moderate COVID-19 received this treatment (Pearson’s Χ^2^ = 12.528; *p* = 0.006). Severe and critical COVID-19 disease is associated with increased plasma concentrations of IL-6 and IL-10 [5], which explain the findings shown in Table 4. Moreover, blood samples for this study were taken before dexamethasone and tocilizumab could possibly have had a therapeutic effect.

### 3.2. COVID-19 and Chronic Cardiovascular Co-Morbidities

Eighty-three (78.30%) of the studied patients had at least one co-morbidity, while seventy-three (87.95%, Pearson’s Χ^2^ = 64.978, *p* < 0.001) of these patients had chronic cardiovascular co-morbidities. The most prevalent chronic cardiovascular diseases (CVD) in studied patients are shown in Table 5.

Additionally, COVID-19 patients with chronic CVD had other co-morbidities as well. The most frequent ones were chronic renal diseases (*n* = 19, 26.03%), *diabetes mellitus* (*n* = 17, 23.29%), oncologic and hematologic diseases (*n* = 15; 20.55%), and chronic respiratory diseases (*n* = 12, 16.44%). Less frequently this patient group also had hypothyroidism (*n* = 7, 9.59%), chronic hepatic diseases (*n* = 5, 6.85%), and rheumatic diseases (*n* = 3, 4.11%). The only condition significantly associated with CVD was chronic renal diseases (Fisher’s exact test *p* < 0.001, Phi coefficient of association value 0.314, *p* = 0.001).

To compare the COVID-19 patients without co-morbidities and COVID-19 patients with chronic CVD co-morbidities, analysis was performed considering clinical data (Table 6), routine laboratory results (Table 7), and cytokine analysis results (Table 8).

From the processed data, it was evident that COVID-19 patients with chronic CVD were significantly older and had immunosuppressive conditions; however, they developed viral pneumonia less frequently than COVID-19 patients without co-morbidities. Furthermore, chronic CVD patients required tocilizumab administration significantly less frequently.

During this study, routine laboratory test results were also analyzed, i.e., red blood cell (RBC) count, hemoglobin concentration, white blood cell, neutrophil, lymphocyte, and thrombocyte counts, lactate dehydrogenase (LDH) activity, ferritin concentration, aspartate aminotransferase (AST) and alanine aminotransferase (ALT) activities, neutrophil-lymphocyte ratio, C-reactive protein, procalcitonin, D-dimer, troponin, total bilirubin, urea concentrations, glomerular filtration rate (GFR), sodium, and potassium concentrations. Significant differences between the two groups are shown in Table 7.

Compared to COVID-19 patients without co-morbidities, COVID-19 patients with chronic CVD significantly more frequently had a decreased RBC count and hemoglobin concentration, which could be associated with advanced age and more prevalent co-morbidities (e.g., chronic kidney diseases), which are known to affect erythropoiesis.

Compared to the COVID-19 patients with no co-morbidities, increased LDH, AST, and ALT activities were significantly less frequent in COVID-19 patients with chronic CVD. It is known that these enzymes are widely distributed in different tissues and organs, such as the heart, lungs, liver, kidneys, skeletal muscle, and hematopoietic system. The serum activity of these enzymes depends on the scale of tissue damage. These findings in our study could be explained by the fact that viral pneumonia was less prevalent in COVID-19 patients with cardiovascular co-morbidities, and tissue damage caused by the infection and inflammatory response in lungs and other internal organs was likely lower.

Compared to COVID-19 patients with no co-morbidities, COVID-19 patients with chronic CVD had significantly higher concentrations of troponin and urea, while GFR was significantly lower. These differences are in accordance with the changes in elderly patients with the presence and chronicity of CVD. Furthermore, patients with chronic CVD also had renal impairment, hence the increased urea concentration and decreased GFR.

Other laboratory markers did not significantly differ between these two patient groups.

**Figure 1:**
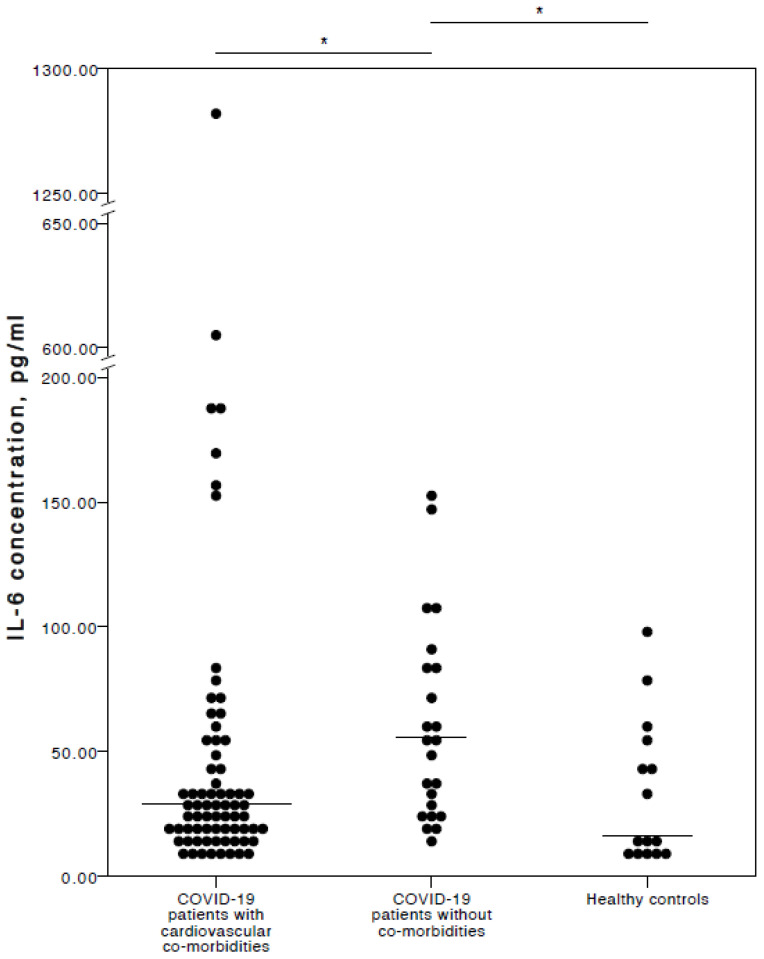
Distribution of plasma IL-6 concentrations in COVID-19 patients with cardiovascular co-morbidities, without co-morbidities, and in healthy controls. Horizontal lines in graphs correspond to medians. * *p* < 0.05.

Interestingly (and in our opinion importantly), the analyses here found that COVID-19 patients with chronic CVD co-morbidities had a significantly lower IL-6 concentration than COVID-19 patients with no co-morbidities (adjusted *p* = 0.021). No significant IL-6 concentration difference was detected between COVID-19 patients with chronic CVD co-morbidities and healthy controls (adjusted *p* = 1.000). Nevertheless, COVID-19 patients with no co-morbidities had significantly higher IL-6 plasma levels than healthy controls (adjusted *p* = 0.018). A visual representation of the IL-6 concentrations is provided in Figure 1. 

### 3.3. COVID-19 and Cardiovascular Medications

Sixty-eight (93.15%, Pearson’s Χ^2^ = 68.488, *p* < 0.001) patients with chronic CVD used at least one drug for the treatment of their particular condition. The median number of medications taken regularly was two (Q_1_ = 0, Q_3_ = 3). These cardiovascular medications are supposed to be consumed on a regular basis prior to being infected by SARS-CoV-2. The most frequently used cardiovascular medications among studied patients are shown in Table 9.

Because of these routine self-medication regimens, this study also explored possible influences of statins and RAASI (angiotensin-converting enzyme inhibitors, angiotensin-II-receptor antagonists, and spironolactone) on COVID-19 patients regarding clinical and routine laboratory testing results. However, the results of the statistical analysis did not reveal any significant differences between chronic CVD COVID-19 patients who took and did not take statins and RAASI.

### 3.4. Cardiovascular Medications and Plasma Cytokine Concentrations in COVID-19 Patients

This study also investigated how statin administration might have potentially affected plasma cytokine levels in COVID-19 patients (Table 10).

**Figure 2:**
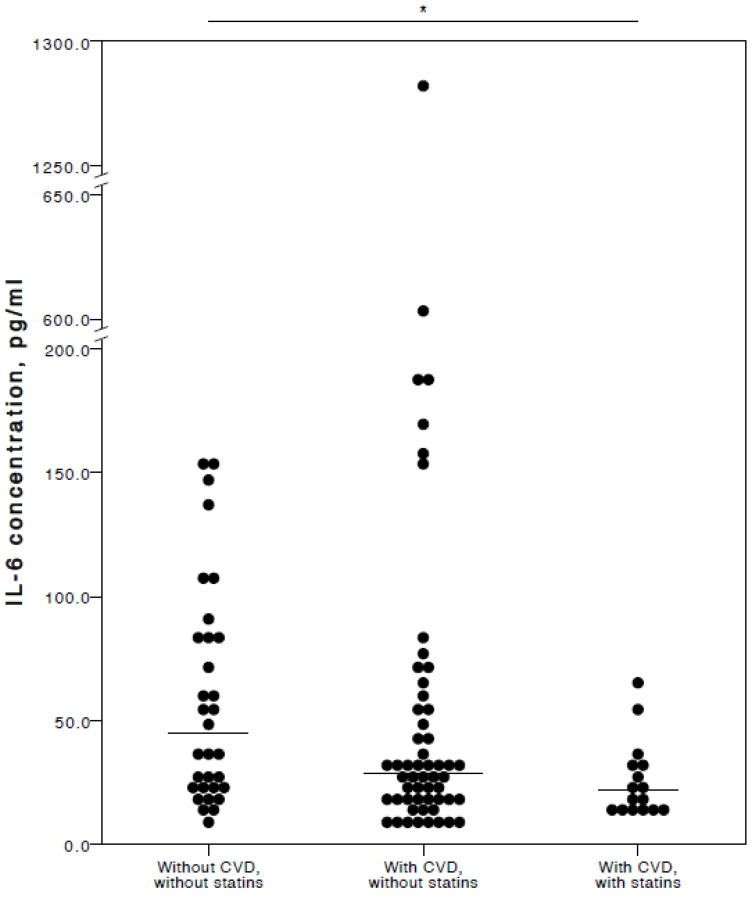
Distribution of plasma IL-6 concentrations in COVID-19 patients without chronic CVD, with chronic CVD but without statin administration, and with both chronic CVD and statin administration. Horizontal lines in graphs correspond to medians. * *p* < 0.05.

**Figure 3:**
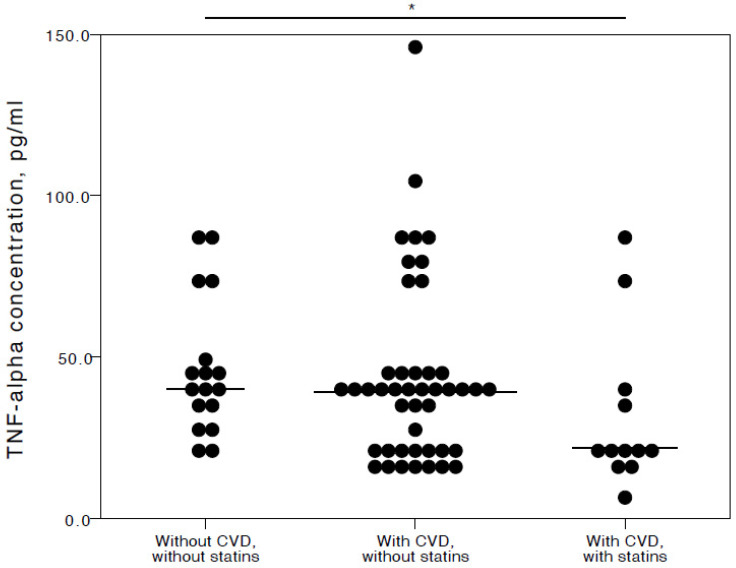
Distribution of plasma TNFα concentrations in COVID-19 patients without chronic CVD, with chronic CVD but without statin administration, and with both chronic CVD and statin administration. Horizontal lines in graphs correspond to medians. * *p* < 0.05.

**Figure 4:**
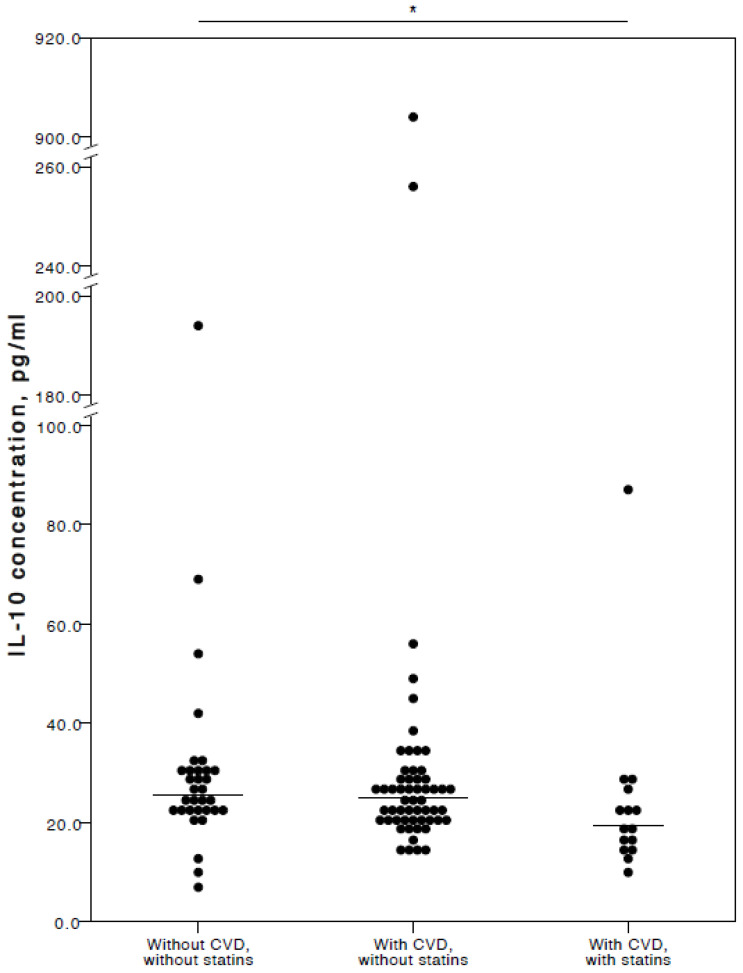
Distribution of plasma IL-10 concentrations in COVID-19 patients without chronic CVD, with chronic CVD but without statin administration, and with both chronic CVD and statin administration. Horizontal lines in graphs correspond to medians. * *p* < 0.05.

The analyses showed that COVID-19 patients with cardiovascular co-morbidities who also consumed statins had significantly lower plasma levels of IL-6 (adjusted *p* = 0.027), TNFα (adjusted *p* = 0.036), and IL-10 (adjusted *p* = 0.025) compared to COVID-19 patients with no CVD co-morbidities. Especially with respect to TNFα and IL-10, it was evident their concentrations were similar in both the COVID-19 patient groups with no cardiovascular co-morbidities and those with cardiovascular co-morbidities without statin use (Figure 2, Figure 3 and Figure 4).

A binary logistic regression model was applied to our data regarding the COVID-19 patients with chronic CVD using statins and COVID-19 patients with no cardiovascular co-morbidities, with statistically significant differences in IL-6, TNFα, and IL-10 concentrations. Although the model appeared to fit the data well, none of these variables showed statistical significance. In an attempt to improve the model, finally, only IL-6 concentration was chosen as a significant variable. An improved binary logistic regression model fit the data well: omnibus test Χ^2^ = 10.026, *p* = 0.02; Hosmer–Lemeshow test Χ^2^ = 6.834, *p* = 0.555; Nagelkerke R^2^ = 0.262. Based on IL-6 concentrations, COVID-19 patients with no co-morbidities were correctly classified in 81.3%, while CVD patients with statin consumption were correctly classified in 43.8% of cases. Wald statistics (5.143, *p* = 0.023) showed that IL-6 was a significant variable, with an OR value of 0.961 (95% CI from 0.929 to 0.995).

Regarding co-administration of statins and medication for COVID-19 treatment, only dexamethasone was significantly less frequently administered in chronic CVD patients regularly using statins. Remdesivir and tocilizumab administration did not differ between these two patient groups. For comparison, dexamethasone was administered in 46/57 (80.7%) patients with chronic CVD who did not use statins, and in 9/16 (56.25%) patients with chronic CVD who used statins (Pearson’s Χ^2^ = 4.021, *p* = 0.045).

The effects of RAASI administration on cytokine concentrations in COVID-19 patients are summarized in Table 11.

**Figure 5:**
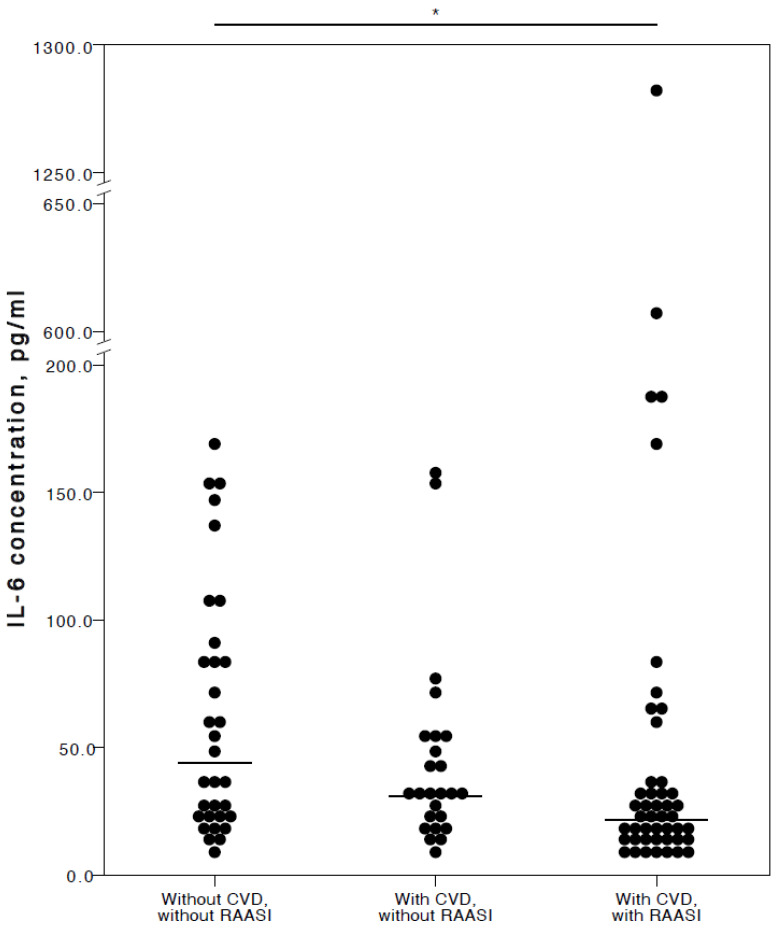
Distribution of plasma IL-6 concentrations in COVID-19 patients without chronic CVD, with chronic CVD but without RAASI administration, and with both chronic CVD and RAASI administration. Horizontal lines in graphs correspond to medians. * *p* < 0.05.

Regarding RAASI, only IL-6 (adjusted *p* = 0.017) was seen to be significantly lower in the plasma of COVID-19 patients with CVD co-morbidities consuming these medications compared to patients who did not have any CVD (Figure 5).

Similarly, a binary logistic regression model was performed to evaluate IL-6 association in COVID-19 patients with no co-morbidities and CVD patients with RAASI consumption. However, the binary logistic model was not suitable for our data set, and IL-6 did not prove to be a statistically significant variable.

Regarding co-administration of RAASI and medication for COVID-19 treatment, only tocilizumab was marginally less frequently administered in chronic CVD patients regularly using RAASI. For comparison, tocilizumab was administered in 4/26 (15.38%) patients with chronic CVD who did not use RAASI, and in 1/47 (2.13%) patients with chronic CVD who used RAASI (Fisher’s exact test *p* = 0.051).

## 4. Discussion

In this study, it was found that COVID-19 patients with chronic CVD and continued use of statins or RAASI had lower plasma concentrations of IL-6 than COVID-19 patients with no co-morbidities, despite their more advanced age and more prevalent immunosuppressive conditions. Anti-inflammatory COVID-19 treatment seemingly did not have a significant influence on such findings. In fact, COVID-19 patients with cardiovascular co-morbidities had IL-6 levels comparable with those in healthy individuals. However, statin or RAASI use did not significantly affect the rates of clinical COVID-19 outcomes in these patients, such as disease severity, supplemental oxygen need, rates of sepsis, septic shock, or death.

Anti-inflammatory properties of statins and RAASI have been explored by researchers not only in respiratory infections but also in other infectious and chronic inflammatory diseases. On the other hand, several investigators have reported an increased risk of severe COVID-19 in patients receiving RAASI; however, a larger proportion of the studies confirmed no association with COVID-19 unfavorable outcomes or even protective action of RAASI against the more severe clinical course of the disease [6].

An experiment in 2014 showed that statins not only impart an anti-inflammatory effect but they are also capable of inducing anti-viral actions. In that study, Mehrbod et al. demonstrated the ability of simvastatin to block the interaction between canine kidney cells and influenza A virus. As a result, influenza A virus-infected cells treated with simvastatin expressed significantly lower levels of TNFα, IL-6, and IFNγ [13]. More recently, Fiore et al. performed cell culture experiments with angiotensin-converting enzyme (ACE)-2-expressing human cells and showed that atorvastatin and pravastatin reduced the secretion of inflammatory mediator p-NF-κB and IL-6, as well as other cytokines, after the cells were stimulated with lipopolysaccharide. Those authors concluded that the data sustained the potential efficacy of statins for use in counteracting or preventing the inflammatory over-formation that is normally triggered by SARS-CoV-2 infection [14]. Building on that premise, Andrianto et al. isolated peripheral blood mononuclear cells, stimulated them with SARS-CoV-2 spike antigen, and then treated them with simvastatin. Though the treatment with statin reduced the expression of IL-6, the finding was not significant [15].

There are not many clinical studies regarding the adjuvant statin use in SARS-CoV-2 infection or COVID-19 disease. A case-control study was performed to evaluate the protective effect of lovastatin in intensive care unit patients with COVID-19. The authors found that IL-6 concentrations were significantly lower in the blood of the patients receiving statin treatment for 1 week as compared to in a non-medicated control group. However, IL-6 concentrations in the blood of patients receiving different doses of lovastatin (20 and 40 mg/day) did not differ [16]. Similarly, a clinical trial was performed to evaluate the effects of statins and aspirin in patients hospitalized with SARS-CoV-2 infection. Among the secondary outcomes, a change in blood IL-6 levels was noted. In patients treated with atorvastatin, there was a 27.83% reduction in IL-6 levels; an even greater reduction (i.e., 53.46%) was achieved with a combination of atorvastatin and aspirin [17].

Regarding bacterial respiratory infections, statin therapy seems to be protective as well. A prospective observational study conducted by Yu et al. revealed that statin administration at least 1 month before the onset of acute ischemic stroke was associated with decreased mortality of ventilator-associated pneumonia and significantly lower serum concentrations of IL-6 and TNFα [18]. However, older studies reported the opposite results. For example, Yende et al. performed a large, prospective, multi-center study of patients hospitalized with community-acquired pneumonia but did not find any evidence of a protective effect for prior or continued statin use. Statin therapy did not significantly improve patient mortality and the risk of severe sepsis, and levels of inflammatory markers and their dynamics were similar between the patients with and without statin use [19]. Another randomized, double-blind, placebo-controlled clinical trial also did not find that adjuvant simvastatin therapy was beneficial in reducing hospitalization duration and inflammatory cytokine (IL-6, IL-10, and TNFα) levels in patients with community-acquired pneumonia. However, those authors noted that the patient samples in both intervention and placebo groups were small and that larger studies were needed to determine any precise roles for statins in community-acquired pneumonia [20].

The role of RAASI in suppressing the inflammation in COVID-19 is potentially supported by an experiment where human epithelial cells were infected by SARS-CoV-2. As a result, the expression of ACE2 was inhibited, leading to increased angiotensin II and angiotensin II type 1 (AT1) receptor expression. This observation was consistent with the literature data wherein SARS-CoV-2 infected patients had higher levels of angiotensin II in their plasma, and these correlated with viral load and lung injury. This experiment also showed that AT1 signaling induced transcriptional regulatory molecules (NF-κB, c-FOS, and MAPK) which, in turn, led to increases in IL-6 secretion by immune cells. Addition of the AT1 receptor antagonist (candesartan) was shown to down-regulate IL-6 secretion through these pathways [21]. On the other hand, a different cell culture study was performed using primary bronchial epithelial cells retrieved from pediatric and adult patients and differentiated ex vivo. Cell cultures were treated with captopril and losartan and then infected by SARS-CoV-2. It was found that neither captopril nor losartan significantly changed ACE2 and IL-6 expression as compared to infected cells without RAASI treatment. In addition, SARS-CoV-2 viral copy numbers were lower in RAASI-treated cell cultures but the differences were not statistically significant [22].

Regarding clinical studies, there is evidence supporting the benefit of RAASI use in patients diagnosed with COVID-19 with pre-existing chronic cardiovascular diseases. In a large single-center retrospective study, patients treated with the RAASI group anti-hypertensive medication had significantly lower IL-6 blood levels compared to in-patients taking other antihypertensive agents. Several other inflammation and coagulation markers and mortality rates were also lower in the RAASI group [23]. In 2021, Cremer et al. performed a retrospective study of the largest COVID-19 patient registry in Europe to analyze the effects of RAASI use in COVID-19 patients with pre-existing cardiovascular conditions. The study authors found that angiotensin receptor blocker use was associated with a significantly improved COVID-19 outcome, in part due to lower circulating levels of mortality-predicting markers, including leukocytes, neutrophils, C-reactive protein, procalcitonin, and IL-6. These patients also had lower levels of thrombogenic activation. In COVID-19 patients, the association between angiotensin receptor blocker intake and mortality appeared to be accompanied by reduced cardiac damage and ameliorated thrombo-inflammatory responses after SARS-CoV-2 infection [24].

In a multi-center retrospective cohort study, patients who used RAASI for the treatment of chronic cardiovascular diseases were found overall to have significantly lower blood levels of several cytokines, including IL-6. The rate of unfavorable outcomes was not different between the patients with RAASI administration and the control group. However, patients taking RAASI had significantly longer lengths of stay in a hospital as well as a prolonged duration of viral shedding [25]. Another study of hypertensive patients showed that patients taking AT1 receptor blockers had significantly lower IL-6 concentrations than patients using other anti-hypertensive agents [26]. A retrospective study of patients with chronic cardiovascular diseases and confirmed COVID-19 disease who were hospitalized in both therapeutic and intensive care units confirmed that circulating levels of several inflammatory markers, including IL-6, were significantly lower in patients who took RAASI; however, this treatment did not significantly affect such unfavorable outcomes as acute respiratory distress syndrome, sepsis, and death [27].

A prospective cohort study found that ACE2 inhibitors were effective in reducing inflammation and, consequently, IL-6 only in patients with ferritin levels that were <1500 µg/L. In patients with hyper-ferritinemia (>1500 μg/L), ACE2 inhibitors were ineffective in reducing IL-6 levels [28]. Nevertheless, several other clinical studies have not shown that the use of RAASI would decrease IL-6 concentrations significantly in patients with COVID-19 and cardiovascular co-morbidities [29,30,31].

Based upon all the available literature, though the findings regarding anti-inflammatory benefits of statins and RAASI are equivocal, there is evidence they might reduce the concentrations of inflammatory markers and improve the outcomes of COVID-19 disease and other respiratory infections. This is especially relevant in patients with chronic cardiovascular co-morbidities who are considered at risk for unfavorable outcomes of respiratory infections.

## 5. Conclusions

COVID-19 patients who had chronic cardiovascular co-morbidities and administered statins or renin–angiotensin–aldosterone system inhibitors had significantly lower plasma concentrations of IL-6 than COVID-19 patients who did not have any co-morbidities. These findings suggest that the use of statins or renin–angiotensin–aldosterone system inhibitors may be of value in COVID-19 patients.

Still, the present study does have inherent limitations. Specifically, in conducting this study, there was little engagement with the patients—hence, the participant number was low. Other limitations included lacking baseline information on the chronic cardiovascular conditions of these patients covering the cardiac function, concentrations of natriuretic peptide (NT-proBNP) and other heart failure markers, body mass index, or lipid panel results, which could be correlated with the cytokine concentrations. Further large-scale studies are needed to clarify the anti-inflammatory effects of statins and RAASI in patients with acute viral respiratory infections such as COVID-19 disease.

## Figures and Tables

**Table 1:** Descriptions of COVID-19 clinical severity groups [11,12].

COVID-19 Severity	Clinical Syndrome	Symptoms and Signs of COVID-19
Mild illness	Uncomplicated upper respiratory tract infection	Non-specific symptoms (fever, cough, sore throat, malaise, headache, muscle ache, nausea, vomiting, diarrhea, loss of smell and taste sensations) with no dyspnea and no changes in chest imaging.
Moderate illness	Pneumonia	Pneumonia with no signs of severe illness [oxygen saturation (SpO_2_) ≥ 94% with ambient air].
Severe illness	Severe pneumonia	Pneumonia with one or more signs of the following: respiratory rate > 30 times/min.; SpO_2_ < 94% with ambient air; ratio of arterial partial pressure of oxygen to fraction of inspired oxygen (PaO_2_/FiO_2_) < 300 mmHg; lung infiltration > 50%.
Critical illness	Acute respiratory distress syndrome (ARDS), sepsis, septic shock, multiple organ dysfunction syndrome (MODS)	Defined by criteria for ARDS, sepsis, septic shock, or other conditions that would require life-sustaining therapies (mechanical ventilation, vasopressor therapy).

**Table 2:** COVID-19 severity in studied patients (*N* = 106).

COVID-19 Severity	Frequency (*n*)	Percentage (%)
Mild	6	5.66
Moderate	27	25.47
Severe	59	55.66
Critical	14	13.21

**Table 3:** Plasma IL-6 and IL-10 levels in recovered and deceased patients with COVID-19.

Cytokines (pg/mL)	Analyte (Median; Q_1_–Q_3_)		Mann–Whitney U Test Value	*p*-Value
	Recovered COVID-19 Patients	Deceased COVID-19 Patients		
	*N* = 89	*N* = 17		
IL-6	27; 17–50	60; 34.5–162.5	1142	<0.001
IL-10	23; 20–29	30; 22.5–39.5	1004	0.020

**Table 4:** Plasma IL-6 and IL-10 levels in patients given dexamethasone and tocilizumab.

Cytokines (as pg/mL)	Analyte (Median; Q_1_–Q_3_)		Mann–Whitney U Test Value	*p*-Value
	No Dexamethasone Administration	With Dexamethasone Administration		
	*N* = 24	*N* = 82		
IL-6	21.75; 15.25–55	31; 20–63.75	1212.5	0.051
IL-10	20.75; 18–25.75	26; 22–30	1312.5	0.006
	**No tocilizumab administration**	**With tocilizumab administration**		
	***N* = 94**	***N* = 12**		
IL-6	27.75; 17–58.63	54.5; 32.25–81.25	760.5	0.034

**Table 5:** The most prevalent chronic cardiovascular diseases in studied patients.

Disease	Frequency (*n*)	Percentage (%) in Cardiovascular Diseases Group	Percentage (%) in All Co-Morbidities Group	Percentage (%) in Total Patient Group
		*N* = 73	*N* = 83	*N* = 106
Arterial hypertension	70	95.89	84.34	66.04
Hypertensive heart disease	41	56.16	49.40	38.68
Ischemic heart disease	18	24.66	21.69	16.98
Heart failure	13	17.81	15.66	12.26
Atrial fibrillation	7	9.59	8.43	6.60
Dyslipidemia	6	8.22	7.23	5.66

**Table 6:** Comparison of clinical characteristics between COVID-19 patients without co-morbidities and patients with chronic cardiovascular diseases.

Characteristic	COVID-19 Patients Without Co-Morbidities	COVID-19 Patients with Cardiovascular Co-Morbidities	Statistic Test Value	*p*-Value
	*N* = 23	*N* = 73		
Males (*n*; %)	16; 69.57	47; 64.38	Pearson’s Χ^2^ = 0.208	0.648
Age, years (median; Q_1_–Q_3_)	53.49; 33.29–58.51	63.78; 52.08–73.45	Mann–Whitney U = 1287	<0.001
Age above 65 years (*n*; %)	1; 4.35	33; 45.21	Fisher’s exact test	<0.001
Immunosuppression (*n*; %)	1; 4.35	22; 30.14	Fisher’s exact test	0.011
Vaccination (*n*; %)	1; 4.35	9; 12.33	Fisher’s exact test	0.423
Days from vaccination to COVID-19 disease onset (median; Q_1_–Q_3_)	53; n/a	104; 54–170	Mann–Whitney U = 2	0.600
Days from symptom onset to hospitalization (median; Q_1_–Q_3_)	8; 4–10	6; 1–11	Mann–Whitney U = 751	0.445
Hospital stay, days (median; Q_1_–Q_3_)	10; 5–17	7; 5–10	Mann–Whitney U = 638.5	0.083
Mild–moderate COVID-19 (*n*; %)	6; 26.09	26; 35.62	Pearson’s Χ^2^ = 1.983	0.371
Severe COVID-19 (*n*; %)	12; 52.17	39; 53.42		
Critical COVID-19 (*n*; %)	5; 21.74	8; 10.96		
COVID-19 as a primary diagnosis (*n*; %)	21; 91.3	61; 83.56	Fisher’s exact test	0.507
Viral pneumonia (*n*; %)	21; 91.3	51; 69.86	Fisher’s exact test	0.052
Bilateral pulmonary infiltration in imaging studies (*n*; %)	16; 69.57	45; 61.64	Pearson’s Χ^2^ = 2.256	0.324
Respiratory insufficiency (*n*; %)	16; 69.57	45; 61.64	Pearson’s Χ^2^ = 0.474	0.491
Supplementary oxygen need (*n*; %)	18; 78.26	53; 72.6	Fisher’s exact test	0.786
Mechanical ventilation (*n*; %)	5; 21.74	9; 12.33	Fisher’s exact test	0.312
Sepsis (*n*; %)	6; 26.09	9; 12.33	Pearson’s Χ^2^ = 2.511	0.113
Septic shock (*n*; %)	5; 21.74	7; 9.59	Fisher’s exact test	0.152
Death (*n*; %)	5; 21.74	11; 15.07	Fisher’s exact test	0.523
Bacterial superinfection (*n*; %)	6; 26.09	13; 17.81	Pearson’s Χ^2^ = 0.755	0.385
Remdesivir administration (*n*; %)	16; 69.57	41; 56.16	Pearson’s Χ^2^ = 1.302	0.254
Dexamethasone administration (*n*; %)	19; 82.61	55; 75.34	Fisher’s exact test	0.577
Tocilizumab administration (*n*; %)	7; 30.43	5; 6.85	Fisher’s exact test	0.007

**Table 7:** Comparison of routine laboratory test results between COVID-19 patients without co-morbidities and patients with chronic cardiovascular diseases.

Characteristics	COVID-19 Patients Without Co-Morbidities	COVID-19 Patients with Cardiovascular Co-Morbidities	Statistic Test Value	*p* Value
	*N* = 23	*N* = 73		
Decreased red blood cell count (*n*; %)	6; 26.09	38; 52.05	Pearson’s Χ^2^ = 7.286	0.026
Decreased hemoglobin concentration (*n*; %)	8; 34.78	45; 61.64	Pearson’s Χ^2^ = 5.787	0.055
Increased lactate dehydrogenase activity (*n*; %)	18; 78.26	39; 53.42	Fisher’s exact test	0.014
Increased aspartate aminotransferase activity (*n*; %)	12; 52.17	18; 24.66	Pearson’s Χ^2^ = 6.990	0.008
Increased alanine aminotransferase activity (*n*; %)	13; 56.52	26; 35.62	Pearson’s Χ^2^ = 4.061	0.044
Troponin concentration, μg/L (median; Q_1_–Q_3_)	0.02; 0.02–0.03	0.04; 0.02–0.05	Mann–Whitney U = 824	0.012
Urea concentration, mmol/L (median; Q_1_–Q_3_)	5.7; 4.2–6.5	7.5; 6.1–13.25	Mann–Whitney U = 1257	<0.001
Glomerular filtration rate, mL/min./1.73m^2^ (median; Q_1_–Q_3_)	102.3; 95.4–119.4	79; 42.45–99.65	Mann–Whitney U = 331.5	<0.001

**Table 8:** Comparison of cytokine results between healthy controls, COVID-19 patients without co-morbidities, and COVID-19 patients with chronic cardiovascular diseases.

Cytokines (pg/mL)	Analyte (Median; Q_1_–Q_3_)			Kruskal–Wallis Test Value	*p*-Value
	Healthy Controls	COVID-19 Patients Without Co-Morbidities	COVID-19 Patients with Cardiovascular Co-Morbidities		
	*N* = 15	*N* = 23	*N* = 73		
IFNβ	126; 104–158	107; 93–136	122; 94–136.5	1.999	0.368
IL-1α/IL-F1	212; 201–216.5	216; 201–226.75	234; 211–238	3.073	0.215
IL-6	13; 10.5–56 ^a^	53; 25–85 ^b^	26.5; 17–47 ^a^	9.594	0.008
IL-12/IL-23 p40	125; 120.5–141.5	134; 125–142	128.25; 125–142	1.498	0.473
TNFα	43; 29–43.5	39.5; 27.5–46	38; 20.13–45	1.317	0.518
IFNγ	124; 120–136	138; 120–156	137; 124–142.5	4.551	0.103
IL-2	102; 84–131	102; 96–114	98; 89.5–102	5.194	0.075
IL-10	20; 17–23.25	25; 22.5–30	23; 20–28	5.554	0.062
Lipocalin-2/NGAL	4125; 3455.25–4693.75	4355; 3655–4874	4399; 4013.5–5173	3.630	0.163

^a,b^ Significant differences between compared groups.

**Table 9:** Most frequently used cardiovascular medications among studied patients.

Cardiovascular Medication	Frequency (*n*)	Percentage (%) in Cardiovascular Diseases Group	Percentage (%) in Total Patient Group
		*N* = 73	*N* = 106
β-adrenergic receptor antagonists	54	73.92	50.94
Renin–angiotensin–aldosterone system inhibitors (RAASI)	48	65.75	45.28
Calcium channel blockers	28	38.36	26.42
Diuretics	25	34.25	23.58
Centrally acting anti-hypertensive drugs	17	23.29	16.04
Statins	17	23.29	16.04
Anti-platelet drugs	13	17.81	12.26

**Table 10:** Comparing cytokine results between COVID-19 patients without chronic CVD and without statin administration, with CVD and without statin administration, and with both CVD and statin administration.

Cytokines (pg/mL)	Analyte (Median; Q_1_–Q_3_)			Kruskal–Wallis Test Value	*p*-Value
	Without CVD, Without Statins	With CVD, Without Statins	With CVD, with Statins		
	*N* = 32	*N* = 57	*N* = 16		
IFNβ	122; 94–137.86	122; 104–135.25	104; 93–150.75	0.022	0.989
IL-1α/IL-F1	234; 215.25–237.5	234; 214.5–238	224; 200.5–234.5	1.910	0.385
IL-6	44; 22–85 ^a^	28.5; 18–56 ^a,b^	21; 15.25–33.75 ^b^	7.223	0.027
IL-12/IL-23 p40	142; 126.25–142	131.5; 125–142	125.5; 125–147.25	1.534	0.464
TNFα	39.5; 31.5–60.5 ^a^	38.5; 21.25–45 ^a,b^	21; 17.13–37.75 ^b^	6.394	0.041
IFNγ	137.5; 124–142.38	137; 124–142.5	134; 121–140	0.353	0.838
IL-2	101.5; 96.5–114	98; 89.5–102.25	97.5; 80.63–102	5.050	0.080
IL-10	25.5; 22.13–30 ^a^	25; 21–29.38 ^a,b^	19; 15–27 ^b^	7.063	0.029
Lipocalin-2/NGAL	4190.5; 3655–4805.5	4325; 4003–5111	4651; 4080.25–6002.25	4.325	0.115

^a,b^ Significant differences between compared groups.

**Table 11:** Comparison of cytokine results between COVID-19 patients without chronic CVD and without RAASI administration, with CVD and without RAASI administration, and with both CVD and RAASI administration.

Cytokines (pg/mL)	Analyte (Median; Q_1_–Q_3_)			Kruskal–Wallis Test Value	*p*-Value
	Without CVD, Without RAASI	With CVD, Without RAASI	With CVD, With RAASI		
	*N* = 32	*N* = 26	*N* = 47		
IFNβ	122; 94–137.88	112; 93.38–136.63	122; 104–136.5	0.684	0.711
IL-1α/IL-F1	234; 215.25–237.5	227.75; 201–237.38	234; 214–238	0.408	0.815
IL-6	44; 22–89.5 ^a^	31; 21–54.25 ^a,b^	22.5; 14.75–35.75 ^b^	7.927	0.019
IL-12/IL-23 p40	134; 125–142	126; 125.25–142	134; 125–142	0.398	0.820
TNFα	39.5; 31.5–73.5	36.5; 19.25–42.5	38; 21–45	3.169	0.205
IFNγ	138; 124–144.38	138; 126.25–158	135.5; 124–140	1.571	0.456
IL-2	102; 98–114	97; 87.5–102.13	98; 89.5–102	4.979	0.083
IL-10	26.5; 22.63–30	24.5; 20–29.63	22.5; 19.25–27.5	4.916	0.086
Lipocalin-2/NGAL	4376; 3655–4988	4402.5; 3883.5–5483.75	4399; 4089–5128	2.278	0.320

^a,b^ Significant differences between compared groups.

## Data Availability

The data presented in this study are available on request from the corresponding author.
